# A protein network refinement method based on module discovery and biological information

**DOI:** 10.1186/s12859-024-05772-z

**Published:** 2024-04-20

**Authors:** Li Pan, Haoyue Wang, Bo Yang, Wenbin Li

**Affiliations:** 1 Hunan Institute of Science and Technology, Yueyang, 414006 China; 2 Hunan Engineering Research Center of Multimodal Health Sensing and Intelligent Analysis, Yueyang, 414006 China

**Keywords:** Protein–protein interaction network, Refined network, Identification of essential proteins, Module discovery

## Abstract

**Background:**

The identification of essential proteins can help in understanding the minimum requirements for cell survival and development to discover drug targets and prevent disease. Nowadays, node ranking methods are a common way to identify essential proteins, but the poor data quality of the underlying PIN has somewhat hindered the identification accuracy of essential proteins for these methods in the PIN. Therefore, researchers constructed refinement networks by considering certain biological properties of interacting protein pairs to improve the performance of node ranking methods in the PIN. Studies show that proteins in a complex are more likely to be essential than proteins not present in the complex. However, the modularity is usually ignored for the refinement methods of the PINs.

**Methods:**

Based on this, we proposed a network refinement method based on module discovery and biological information. The idea is, first, to extract the maximal connected subgraph in the PIN, and to divide it into different modules by using Fast-unfolding algorithm; then, to detect critical modules according to the orthologous information, subcellular localization information and topology information within each module; finally, to construct a more refined network (CM-PIN) by using the identified critical modules.

**Results:**

To evaluate the effectiveness of the proposed method, we used 12 typical node ranking methods (LAC, DC, DMNC, NC, TP, LID, CC, BC, PR, LR, PeC, WDC) to compare the overall performance of the CM-PIN with those on the S-PIN, D-PIN and RD-PIN. The experimental results showed that the CM-PIN was optimal in terms of the identification number of essential proteins, precision-recall curve, Jackknifing method and other criteria, and can help to identify essential proteins more accurately.

## Background

Proteins are the most significant components of living organisms and have very important biological functions, participating in gene regulation, cellular metabolism, and are the main bearers of biological life activities. Proteins are subdivided into essential and non-essential proteins, among which, essential proteins are particularly important for life activities, and their absence can lead to the failure of the organism to survive [1]. In addition, essential proteins are associated with human disease-causing genes, and their identification and analysis can help in the design of drug targets.

Early studies of essential proteins were mainly conducted by wet experimental methods such as RNA interference [2], single gene knockout [3] and conditional gene knockout [4], which often have the drawbacks of being expensive and time-consuming, therefore, the identification of essential proteins by computational methods has become the current trend.

Node ranking methods are commonly used to identify essential proteins in the protein–protein interaction network (PIN). Initially, researchers used network-based centrality methods to identify essential proteins in the original PIN (static PIN) [5], such as degree centrality (DC) [6], local average connectivity centrality (LAC) [7], node clustering centrality (NC) [8], maximum neighborhood component density centrality (DMNC) [9], topological potential centrality (TP) [10], neighbor interaction density centrality (LID) [11], closeness centrality (CC) [12], betweenness centrality (BC) [13], pagerank centrality (PR) [14], leaderrank centrality (LR) [15], etc.

However, the centrality methods only use the topological features of protein interaction networks for assessing the importance of proteins, and thus it’s difficult to obtain desired predictive performance. In recent years, researchers tended to integrate multiple biological information of proteins to help identify essential proteins more accurately. For example, Li [16] et al. and Tang [17] et al., proposed the PeC and the WDC methods by integrating the degree of co-expression between protein pairs in gene expression profiles and the edge clustering coefficients of their interactions. Qin et al. [18] proposed the LBCC method, which is based on network topological features and protein complex; Li et al. [19] pointed out that proteins in complex are more likely to be essential than proteins not present in the complex, and they proposed the UC method by combining protein complexes and topological features of PINs. Lei et al. [20] proposed the PCSD method that fuses the degree of protein complex involvement and subgraph density. Zhong et al. [21] used a dynamic threshold method to binarize gene expression values and proposed the JDC method to combine the co-expression states and edge clustering coefficients of protein pairs at multiple times.

Although these node ranking methods have made great progress in identifying essential proteins, most of them require the use of topological information of proteins in the PIN for identification of essential proteins, especially network-based centrality methods, which are highly dependent on the accuracy of the underlying PINs. However, most of the PINs obtained from high-throughput experiments have been found to contain false positives or false negatives [22], which may somewhat interfere with the identification accuracy of essential proteins by most node ranking methods.

To improve the identification accuracy of essential proteins, some researchers used biological information of proteins to filter out unreliable interactions between proteins in the PIN, thereby constructing a refined PIN to identify essential proteins for node ranking methods. For example, based on static PIN (S-PIN), Xiao et al. [23] removed from it some unreliable interactions by determining whether protein pairs were activated at the same time in terms of gene expression level data, and constructed a once-refined PIN (D-PIN). Subsequently, Li et al. [24] further removed some unreliable interactions from the DPIN by determining whether protein pairs appeared in the same subcellular compartment, and constructed a twice-refined PIN (RD-PIN).

Nevertheless, some researchers pointed out that PINs have modular characteristics [25–27], the essentiality of a protein is not only related to the protein itself, but also to the functional module in which the protein is located, and proteins within modules have higher similarity than those in other modules. Furthermore, Zotenko et al. [28] found that in PINs, a large number of essential proteins may be present in highly dense functional modules. The aforementioned studies focused only on the edges between protein nodes to refine the network, ignoring the modularity feature of PINs. Therefore, it is still a question worth exploring how to better utilize the modularity feature of PINs to construct an efficient PIN and improve the performances of node ranking methods.

For the identification of community structure in complex networks, researchers have proposed a series of module discovery algorithms. For example, algorithms based on modularity [29, 30] and information-theoretic framework [31] can divide non-overlapping modules in complex networks; while the modules discovered by using clique-percolation based [32] and edge-clustering based [33] methods can be overlapping. In particular, in recent studies, some researchers have made use of network structure and node attributes to cluster complex networks more accurately [34–36]. For example, Hu et al. [35] and Yang et al. [36], developed two fuzzy-based graph clustering algorithms that well take into account the key dependencies between node embedding and resulting clustering. In our study, a modularity-based Fast-unfolding algorithm was used to partition PINs into modules and analyze the differences between modules.

We found that the biological and topological information contained in different modules of PIN varies greatly. For example, some modules are dense but contain few essential proteins, which may be counterproductive for identifying essential proteins in the PIN. Therefore, the identification and selection of critical modules is of great significance for the construction of higher quality PINs. That is to say, if the network can be refined properly in combination with the modularity of the PIN, the performance of the node ranking method in the PIN may be improved more effectively.

Based on this, in this paper, we proposed a network refinement method based on module discovery and biological information to improve the identification accuracy of essential proteins for node ranking methods. The idea is, for a PIN, firstly, to remove the interactions in some small connected subgraphs from the PIN; secondly, to divide the maximal connected subgraph into several closely connected modules by the Fast-unfolding algorithm that fuses the modularity; thirdly, to select the critical modules by combining orthologous information and subcellular localization information of proteins and topological features of each module; finally, to construct a more refined PIN (CM-PIN) according to the selected critical modules.

To evaluate the effectiveness of the network refinement method proposed in this paper, two different species of Saccharomyces cerevisiae and Human sapiens were used for validation. We applied 12 node ranking methods (LAC, DC, DMNC, NC, TP, LID, CC, BC, PR, LR, PeC, WDC) on the S-PIN, D-PIN, and RD-PIN, and compared the results with those on the CM-PIN obtained on these networks, respectively. The experimental results showed that in terms of the identification number of essential proteins at top 100–600, Jackknifing method, the area under the precision-recall curves, sensitivity, specificity, positive predictive value, negative predictive value, F-measure, Matthews correlation coefficient and accuracy, the performances of the 12 node ranking methods on the CM-PIN are optimal. All of these prove that the network refinement method proposed in this paper can obtain a more efficient PIN, which is conducive to improve the identification accuracy of essential proteins for node ranking methods, and is superior to the existing refinement networks (D-PIN and RD-PIN).

## Methods

**Fig. 1:**
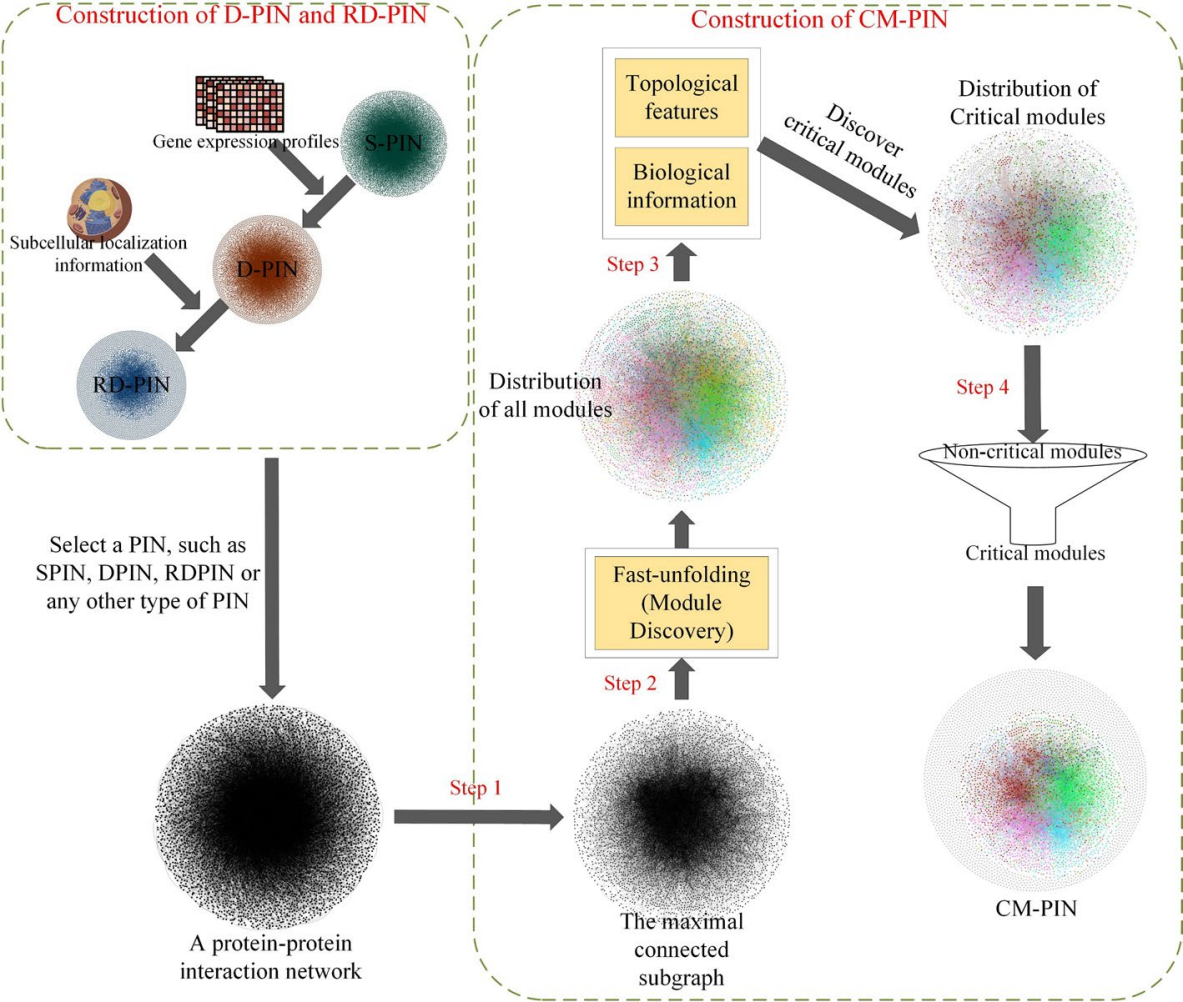
The overall steps of the construction of the CM-PIN. First, in the block of construction of D-PIN and RD-PIN, we combined static PIN (S-PIN) and gene expression profile to construct D-PIN, and then further combined subcellular localization information to construct RD-PIN. In this paper, corresponding CM-PINs will be constructed based on these networks. Secondly, in the block of construction of CM-PIN, the Step 1 is to extract the maximum connected subgraph of a given PIN; the Step 2 is to divide the maximum connected subgraph into several modules using the Fast-unfolding algorithm; and the Step 3 is to identify critical modules using the biological (orthologous information and subcellular localization information) and topological information of proteins; the Step 4 is to refine the given PIN and construct the CM-PIN according to the identified critical modules

In this section, first, we described how to build these three protein interaction networks: S-PIN, D-PIN, and RD-PIN. Second, we described how to screen the critical modules by the biological information of proteins and the topological features of each module, and constructed CM-PINs, on S-PIN, D-PIN, and RDPIN respectively, the overall steps of this approach were shown in Fig. 1.

### S-PIN, D-PIN and RD-PIN

A static protein–protein interaction network (S-PIN) [37–39], is an undirected graph *G*_*S*_ = (*V*_*S*_, *E*_*S*_), where *V*_*S*_ represents the set of proteins and *E*_*S*_ represents the set of protein interactions.

A dynamic protein–protein interaction network (D-PIN) [23] is an edge-induced subgraph *G*_*D*_ = (*V*_*D*_, *E*_*D*_) of the S-PIN in terms of the gene expression levels of proteins, where *V*_*D*_ = *V*_*S*_ and *E*_*D*_ ⊆ *E*_*S*_. Let *e*_*ik*_ denotes the value of gene expression level of *v*_*i*_ at time point *t*_*k*_. If *e*_*ik*_ is greater than *τ*_*i*_, then *v*_*i*_ is active at time point *t*_*k*_. for any (*v*_*i*_, *v*_*j*_) ∈ *E*_*S*_, if both *v*_*i*_ and *v*_*j*_ are activated at time point *t*_*k*_, the interaction between them is preserved in *E*_*D*_, otherwise it is removed from *E*_*D*_. The activity threshold *τ*_*i*_ of protein *v*_*i*_ was calculated by using the following equation [25]:

1 $$\tau_{i} = \mu_{i} + \sigma_{i}$$

2 $$\mu_{i} = \frac{{\sum\nolimits_{k = 1}^{n} {e_{ik} } }}{n}$$

3 $$\sigma_{i} = \sqrt {\frac{{\sum\nolimits_{k = 1}^{n} {(e_{ik} - \mu_{i} )^{2} } }}{n}}$$

where *μ*_*i*_ denotes the mean of the *n* time-point gene expression level values of the protein and *σ*_*i*_ is the standard deviation of the gene expression level values of *v*_*i*_. In this paper, *n* = 36 for Saccharomyces cerevisiae and *n* = 64 for Human sapiens.

A refined dynamic protein–protein interaction network (RD-PIN) [24] is an edge-induced subgraph *G*_*RD*_ = (*V*_*RD*_, *E*_*RD*_) of the D-PIN in terms of subcellular localization information of proteins, where *V*_*RD*_ = *V*_*D*_ and *E*_*RD*_ ⊆ *E*_*D*_. Let *L*(*v*_*i*_) = {*l*_*1*_(*v*_*i*_), …, *l*_*m*_(*v*_*i*_), …, *l*_*r*_(*v*_*i*_)} be the 11 subcellular localization statuses of protein *v*_*i*_, where *r* = 11. If *v*_*i*_ is in the *m*th subcellular compartment, then *l*_*m*_(*v*_*i*_) = 1, otherwise *l*_*m*_(*v*_*i*_) = 0. For any (*v*_*i*_, *v*_*j*_) ∈ *E*_*D*_, only when *l*_*m*_(*v*_*i*_) = *l*_*m*_(*v*_*j*_) = 1, the interaction between *v*_*i*_ and *v*_*j*_ will be preserved in *E*_*RD*_, otherwise their interaction will be removed from the *E*_*RD*_.

### Construction of the CM-PIN

The construction of the CM-PIN consists of four steps (the following steps are consistent with Fig. 1):

- Step 1: retaining interactions in maximal connected subgraphs, that is, to remove the interactions in the remaining small connected subgraphs of the given PIN;
- Step 2: module discovery based on Fast-unfolding algorithm, that is, to divide the obtained maximum connected subgraph into several modules using the Fast-unfolding algorithm;
- Step 3: detecting critical modules,that is, to screen out critical modules by using biological and topological information of modules;
- Step 4: refining the protein–protein interaction network, that is, to remove the interaction of non-critical modules in the original PIN and construct the CM-PIN.

The construction process of the CM-PIN is described in the following algorithm.

**Figure:**
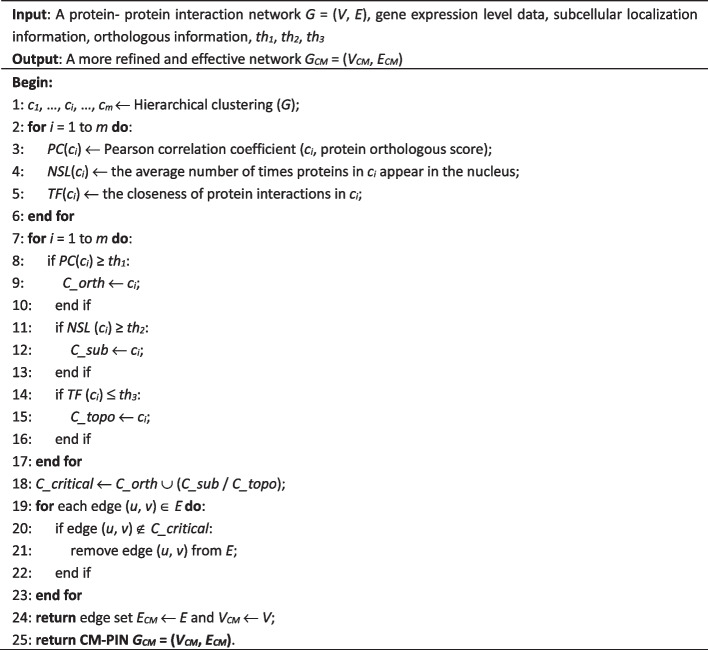
**Algorithm: Construction of the CM-PIN**

#### Step 1: retaining interactions in maximal connected subgraphs

It has been found that PINs have scale-free properties [40, 41]. The scale-free property means that the degrees of the nodes in PIN obey a power-law distribution, so PIN belongs to a scale-free network. Considering that PIN is a disconnected graph and consists of several connected subgraphs, where most of the proteins and their interactions are present in a maximal connected subgraph, while the number of proteins and their interactions in some remaining connected subgraphs are very small. As shown in Table 1, we counted the proportion of interactions in the maximal connected subgraphs of the YDIP, YBioGRID and HDIP datasets to the original network interactions.

**Table 1:** The proportion of interactions in the maximal connected subgraphs to the original network interactions on YDIP, YBioGRID and HDIP datasets

Networks	YDIP	YBioGRID	HDIP
S-PIN	15,123/15,166 = 99.72%	52,832/52,833 = 99.99%	6412/6892 = 93.04%
D-PIN	9436/9514 = 99.18%	32,730/32,735 = 99.98%	3974/4414 = 90.03%
RD-PIN	4953/5175 = 95.71%	18,330/18,362 = 99.83%	2191/2508 = 87.36%

#### Step 2: module discovery based on Fast-unfolding algorithm

It has been shown that PINs have modular properties [25, 26], and the modularity reflects the presence of highly connected protein clusters in PINs. So far, the clustering of protein interaction networks is an effective method for module delineation. In the paper, the Fast-unfolding module discovery algorithm, a hierarchical clustering method, is used for module division of the PIN.

The purpose of module partitioning is to make the connections within the partitioned modules tighter and the connections between modules sparser. In order to evaluate whether the module division is feasible, Newman et al. [29] proposed the concept of modularity. Defining *e*_*ii*_ as the ratio of the sum of all connected edges within module *i* to the total number of edges in the network and *a*_*i*_ as the ratio of the total number of neighboring nodes of nodes within module *i* to the total number of edges, the modularity *Q* can be expressed as:

4 $$Q = \sum\nolimits_{i} {(e_{ii} - a_{i}^{2} )}$$

A larger modularity represents a tighter connection within the module, and conversely, a smaller modularity represents a sparser connection within the module, and when the modularity *Q* reaches its maximum value, the division of modules is optimal.

Blondel et al. [30] proposed a Fast-unfolding algorithm for discovering module structures on large networks, which is a heuristic algorithm based on modularity optimization. Compared with traditional module discovery algorithms, Fast-unfolding has lower time complexity on large-scale networks and stable results for module partitioning, which is the reason why this algorithm is chosen to partition modules in this paper. The implementation steps of Fast-unfolding algorithm are as follows: first, initialization, divide each protein node into different modules; second, for each protein node, try to divide it into the module where its neighboring nodes are located, calculate the modularity *Q* at this time, and judge whether the difference *ΔQ* between the modularity before and after the division is positive, if it is positive, accept this division, if not, abandon this division; third, repeat the above process until the modularity *Q* can no longer be increased, then the division of modules is completed, and *C* = {*c*_*1*_, *c*_*2*_, …, *c*_*i*_, …, *c*_*m*_} is the set of modules and *m* is the number of module divisions. It is worth noting that the divided modules are non-overlapping.

#### Step 3: detecting critical modules

To determine the importance of each module, we used three features (i.e., orthologous information, subcellular localization information, and topological information of the module) to score each module in the PIN.

(1) Determine the importance of modules using orthologous information of proteins.

Studies have shown that essential proteins evolve much more slowly than non-essential proteins [42], i.e., essential proteins are more conserved. We believe that the modules containing more conserved proteins are more likely to be critical, and the conserved properties of proteins can mainly be found in the orthologous information of proteins. Therefore, we calculate the Pearson correlation coefficient between each module and the protein orthologous information in the PIN as the first score of the module. For protein *v*_*i*_, let *O*(*v*_*i*_) represent the set of reference organisms in which at least an orthologous protein pair including *v*_*i*_ occurs, |*O*(*v*_*i*_)| is the orthologous score of *v*_*i*_, and the vector consisting of orthologous scores of all proteins in the PIN is represented by *y*. For a module *c*_*i*_, its vector is represented as *xi* that only contains 0 and 1 (1 if the protein is in the module *c*_*i*_, 0 otherwise). The Pearson correlation coefficient *PC*(*c*_*i*_) between module *c*_*i*_ and the orthologous scores is:

5 $$PC(c_{i} ) = \frac{{\sum\nolimits_{j = 1}^{n} {(xi_{j} - \mu_{xi} )(y_{j} - \mu_{y} )} }}{{\sqrt {\sum\nolimits_{j = 1}^{n} {(xi_{j} - \mu_{xi} )^{2} } \sum\nolimits_{j = 1}^{n} {(y_{j} - \mu_{y} )^{2} } } }}$$

where *n* is the number of proteins in the PIN, and *μ*_*xi*_ and *μ*_*y*_ are the mean values of *xi* and *y*. Thus, the set of possible critical modules selected based on the orthologous information of the proteins within the module is denote as *C_orth* = {*c*_*i*_|*PC*(*c*_*i*_) ≥ *th*_*1*_}, where *th*_*1*_ is a threshold value.

(2) Determine the importance of modules using subcellular localization information of proteins.

The importance of the protein is not only related to the orthologous information of the protein, but also to the subcellular localization information of the protein, which can identify the critical modules in the PIN from another perspective. We observed the number of times proteins and essential proteins were present in each subcellular compartment, and found that proteins and even essential proteins were most widely distributed in the nucleus. Therefore, we thought that the more times proteins within the module were present in the nucleus, the more likely that module was critical. For the module *c*_*i*_, we calculate the number of times the protein in module *c*_*i*_ occurs in the nucleus as its second score, denoted by *NSL*(*c*_*i*_):

6 $$NSL(c_{i} ) = \frac{{N(c_{i} )}}{{n(c_{i} )}}$$

where *N*(*c*_*i*_) is the number of times the protein within the module appears in the nucleus and *n*(*c*_*i*_) is the number of nodes within the module. The set of the possible critical modules selected based on the subcellular localization information of the proteins within the module is represented by *C_sub* = {*c*_*i*_|*NSL*(*c*_*i*_) ≥ *th*_*2*_}, where *th*_*2*_ is a threshold value.

(3) Determine the importance of modules using topological characteristics of modules.

To identify the importance of the module, we also used the topological characteristics of each module in the network. It has been pointed out that a large number of essential proteins may exist in highly dense functional modules [28]. Thus, we thought that the richer the interactions within the module, the more likely it is to play an important role in the whole network, so we calculated the topological characteristics of module *c*_*i*_ as its third score, denoted by *TF*(*c*_*i*_):

7 $$TF(c_{i} ) = \frac{{I(c_{i} ) - O(c_{i} )}}{{n(c_{i} )}}$$

where *I*(*c*_*i*_) is the number of interactions inside module *c*_*i*_, *O*(*c*_*i*_) is the number of interactions between module *c*_*i*_ and other modules, and *n*(*c*_*i*_) is the number of nodes of module *c*_*i*_. And according to the topological characteristics of the module, modules less than *th*_*3*_ are selected as the set of potentially non-critical modules, that is, *C_topo* = {*c*_*i*_|*TF*(*c*_*i*_) ≤ *th*_*3*_}, where *th*_*3*_ is a threshold value.

#### Step 4: refining the protein–protein interaction network

Finally, we integrated the above three features of the modules to obtain the final selected critical modules, that is, *C_critical* = {*c*_*i*_|*C_orth* ∪ (*C_sub*/*C_topo*)}. For a PIN (S-PIN, D-PIN or RD-PIN) *G* = (*V*, *E*), ∀(*v*_*i*_, *v*_*j*_) ∈ *E*, if *v*_*i*_ and *v*_*j*_ are both in the critical modules *C_critical*, their interaction will be retained, otherwise their interactions will be removed from the *E*, thus obtain the finally refined *E*_*CM*_, resulting in a more refined CM-PIN, *G*_*CM*_ = (*V*_*CM*_, *E*_*CM*_), where *V*_*CM*_ = *V*.

## Experiment and discussion

### Materials and datasets

We first performed a complete experiment using the Saccharomyces cerevisiae dataset, as this dataset is currently the most complete of all species and has been widely used to test various methods for identifying essential proteins. Then, we used the Human sapiens dataset to verify the validity of the proposed method.

#### Protein–protein interaction datasets and essential proteins

The two protein–protein interaction datasets from Saccharomyces cerevisiae used in this paper were downloaded from YDIP [43] and YBioGRID [44], which contain 15,166 and 52,833 interactions, respectively, covering 4746 and 5616 proteins. A dataset of protein–protein interactions from Homo sapiens was downloaded from HDIP [45], which contains 6892 interactions covering 4615 proteins. Essential proteins were collected from the following data sets [46–48]: DEG, MIPS, SGD, OGEE. The YDIP, YBioGRID, and HDIP datasets contain 1130, 1199 and 726 essential proteins, respectively.

#### Other biological information

(1) Gene expression profile: The gene expression profiles of the yeast and human datasets were downloaded from GSE3431 [49] and GSE86354 [50], respectively, containing 6,777 and 18,912 proteins. GSE3431 dataset records the observation data of 36 time points during three successive metabolic cycles and GSE86354 dataset records expression profiles across 8 tissue including 64 time points. (2) Subcellular localization information: Subcellular location information for both species was downloaded from the COMPARTMENTS dataset [51], which both contain 11 subcellular compartments. (3) Orthologous information: Information on orthologous proteins of yeast and human was taken from Version 7 [52] and Version 8 [53] of the InParanoid database, which contain 100 and 162 genome-wide paired comparison sets, respectively.

### Node ranking methods

To verify the performance of the CM-PIN, we used 12 typical node ranking methods (DC [6], LAC [7], NC [8], DMNC [9], TP [10], LID [11], CC [12], BC [13], PR [14], LR [15], PeC [16], WDC [17]) and compared their performances of the identification of essential proteins on the CM-PIN with that on the S-PIN and two existing refinement networks (D-PIN [23] and RD-PIN [24]). The node ranking method will first calculate the importance scores of all protein nodes in the network according to its formula, then rank the proteins in descending order according to the importance scores, and finally a part of highly ranked proteins will be considered as essential proteins.

### Experimental results and analysis on Saccharomyces cerevisiae

#### Analysis of the number of essential proteins identification

In order to prove that the network refinement method proposed in this paper can effectively improve the number of essential proteins identified by each node ranking method, we obtained more efficient CM-PINs on the SPIN, DPIN and RDPIN of the YDIP and YBioGRID datasets, respectively. And the numbers of essential proteins identified by node ranking methods at top 100, top 200, top 300, top 400, top 500, and top 600 on the CM-PIN were compared with their performance on the S-PIN, D-PIN, and RD-PIN, as shown in Tables 2 and 3. We denoted CM-PIN refined from S-PIN (D-PIN or RD-PIN) by CM-PIN(S) (CM-PIN(D) or CM-PIN(RD)), and marked the optimal item in bold when comparing two or more items in all subsequent tables.

**Table 2:** Comparison of the number of essential proteins identified by 12 node ranking methods on the S-PIN, D-PIN, RD-PIN and the CM-PIN at top 100–600 on YDIP dataset

Methods	S-PIN	CM-PIN(S)	D-PIN	CM-PIN(D)	RD-PIN	CM-PIN(RD)
LAC	[82,144,195, 251,300,347]	[**82,144,201,** **263,314,366**]	[77,147,202, 271,318,362]	[**81,154,219,** **275,330,377**]	[76,147,207, 264,315,363]	[**83,157,225,** **288,341,391**]
DC	[55,102,152, 202,256,300]	[**66,121,190,** **241,282,337**]	[59,120,172, 227,276,323]	[**73,142,204,** **259,318,363**]	[71,136,200, 259,314,369]	[**80,152,219,** **281,343,398**]
DMNC	[61,116,149, 192,249,292]	[**61,119,163,** **215,274,339**]	[61,131,164, 219,274,316]	[**69,136,185,** **255,308,357**]	[61,120,164, 204,275,338]	[**72,145,199,** **273,330,386**]
NC	[78,143,200, 250,290,337]	[**84,147,208,** **260,309,355**]	[81,141,207, 261,310,342]	[**86,155,215,** **275,320,375**]	[80,145,205, 259,308,346]	[**84,154,226,** **287,339,390**]
TP	[55,106,150, 194,236,282]	[**64,120,179,** **235,280,323**]	[59,118,174, 220,268,310]	[**70,140,205,** **262,314,356**]	[72,134,198, 260,322,372]	[**79,154,221,** **283,341,395**]
LID	[82,142,199, 251,303,347]	[**82,142,206,** **260,316,354**]	[83,149,214, 271,317,364]	[**86,155,226,** **281,337,385**]	[82,151,214, 265,316,371]	[**85,156,227,** **292,353,405**]
BC	[48,83,123, 164,207,247]	[**51,107,160,** **210,253,292**]	[52,93,135, 177,215,250]	[**62,114,165,** **210,259,290**]	[54,110,160, 213,262,305]	[**67,127,189,** **228,280,324**]
CC	[49,90,134, 178,225,263]	[**57,107,159,** **203,256,290**]	[53,103,143, 188,230,264]	[**65,121,173,** **211,266,304**]	[67,120,171, 227,282,339]	[**70,126,179,** **242,304,365**]
PR	[51,94,145, 190,246,291]	[**61,116,178,** **232,277,326**]	[55,103,153, 206,255,303]	[**67,129,187,** **244,295,344**]	[60,110,182, 233,283,327]	[**70,140,205,** **260,315,368**]
LR	[58,104,151, 194,234,269]	[**65,123,173,** **215,255,290**]	[60,108,149, 196,230,265]	[**66,127,177,** **213,254,304**]	[69,125,173, 209,244,291]	[**78,135,181,** **230,277,335**]
PeC	[74,141,202, 250,296,331]	[**83,160,220,** **277,322,359**]	[73,138,198, 250,301,335]	[**85,159,220,** **279,323,364**]	[75,149,199, 255,298,339]	[**85,164,226,** **281,331,369**]
WDC	[76,146,218, 268,321,367]	[**82,158,227,** **282,340,386**]	[74,146,208, 262,316,358]	[**84,161,223,** **289,337,386**]	[80,150,213, 284,328,369]	[**88,166,236,** **303,353,397**]

**Table 3:** Comparison of the number of essential proteins identified by 12 node ranking methods on the S-PIN, D-PIN, RD-PIN and the CM-PIN at top 100–600 on YBioGRID dataset

Methods	S-PIN	CM-PIN(S)	D-PIN	CM-PIN(D)	RD-PIN	CM-PIN(RD)
LAC	[43,104,159, 217,277,325]	[**43,123,189,** **256,314,351**]	[54,100,175, 236,287,334]	[**54,138,209,** **265,316,365**]	[57,103,180, 241,290,339]	[**57,137,215,** **271,323,380**]
DC	[54,99,149, 208,254,298]	[**55,110,181,** **241,295,343**]	[55,108,172, 231,278,310]	[**56,122,189,** **254,295,339**]	[61,128,196, 244,289,341]	[**62,129,205,** **261,317,367**]
DMNC	[25,81,144, 183,225,267]	[**46,107,173,** **227,274,316**]	[34,94,161, 204,251,303]	[**55,126,190,** **237,292,332**]	[28,95,157, 207,253,307]	[**55,132,191,** **237,295,350**]
NC	[42,110,165, 217,270,321]	[**44,130,192,** **257,317,370**]	[53,108,176, 245,299,343]	[**55,135,216,** **274,320,375**]	[56,109,183, 245,300,347]	[**57,140,215,** **278,328,379**]
TP	[48,86,132, 180,218,250]	[**57,108,171,** **227,283,332**]	[45,105,147, 186,221,252]	[**55,115,178,** **237,293,329**]	[70,134,192, 247,302,339]	[**70,136,196,** **249,300,341**]
LID	[43,103,157, 219,276,324]	[**43,122,189,** **259,319,357**]	[55,99,173, 243,287,334]	[**55,137,212,** **269,312,364**]	[57,102,179, 247,293,340]	[**57,138,217,** **273,326,374**]
BC	[47,95,141, 175,204,248]	[**69,129,175,** **232,270,317**]	[47,86,141, 186,222,259]	[**54,110,159,** **209,262,304**]	[41,81,123, 174,216,260]	[**47,89,138,** **190,229,279**]
CC	[39,69,99, 128,161,184]	[**61,104,151,** **197,249,292**]	[31,63,89, 115,140,178]	[**51,97,146,** **193,241,279**]	[50,91,**143**, 193,237,273]	[**50,91,**141**,** **197,239,273**]
PR	[51,106,150, 190,238,286]	[**64,120,177,** **239,286,341**]	[55,105,156, 201,257,298]	[**60,113,176,** **232,285,333**]	[61,121,179, 226,264,307]	[**64,131,183,** **238,278,336**]
LR	[54,99,133, 165,197,226]	[**59,105,157,** **202,241,276**]	[54,99,141, 164,190,223]	[**62,102,141,** **184,222,226**]	[56,112,152, 184,218,253]	[**58,116,161,** **196,232,266**]
PeC	[59,97,172, 232,280,325]	[**61,135,202,** **261,309,352**]	[60,101,174, 230,278,317]	[**61,134,203,** **256,305,341**]	[63,101,176, 235,286,327]	[**63,138,207,** **263,314,354**]
WDC	[53,110,163, 229,283,326]	[**56,126,199,** **262,311,362**]	[56,115,169, 235,283,323]	[**58,131,210,** **263,313,355**]	[60,108,186, 248,292,347]	[**62,135,213,** **273,328,381**]

It can be seen that the CM-PIN can significantly improve the identification accuracy of essential proteins by node ranking methods on yeast datasets, whether it is static PIN or refined PIN, and the values of top 100-top 600 on the CM-PIN are higher than those of the other three existing PINs. Compared with different PINs, the average improvement ratio of 12 node ranking methods at top 600 on YDIP and YBioGRID datasets was: 9.82% and 20.58% for the CM-PIN refined on the S-PIN; 11.30% and 15.15% for the CM-PIN refined on the D-PIN; 9.65% and 7.79% for the CM-PIN refined on the RD-PIN. And even some node ranking methods have a significant improvement, for example, compared with the S-PIN, the BC method has improved by 18.22% at top 600 on the CM-PIN on YDIP dataset; compared with the D-PIN, the CC method has improved by 56.74% at top 600 on the CM-PIN on YBioGRID dataset. In addition, the LID method was able to identify 405 essential proteins at top 600 on the CM-PIN refined on the RD-PIN on YDIP dataset, which has a very high identification accuracy. All of these illustrated the effectiveness of our method and demonstrate that CM-PIN is a more refined and effective network.

It is worth noting that the focus of this paper is to improve the overall performance of node ranking methods, so we pay more attention to the accuracy of these methods at top 1130 for YDIP (top 1199 for YBioGRID, or top 7,26 for HDIP). Meanwhile, the accuracy at top 100 can also receive a certain increase at this case. On the other hand, if we want to focus on the improvement of the performance at the top 100, we can also achieve good results in the accuracy of the top 100 by adjusting the parameters of our method appropriately. For example, when setting the parameters *th*_*1*_ = 0.1, *th*_*2*_ = 2, and *th*_*3*_ = −2, the CM-PIN(RD) for YBioGRID can significantly improve the top 100 values of the node ranking methods. However, their top 1199 values will decline to a certain extent at this time. Therefore, the readers can strengthen the specified performance index by adjusting the parameters according to their own concerns.

#### Validated by using the Jackknifing method

**Fig. 2.**
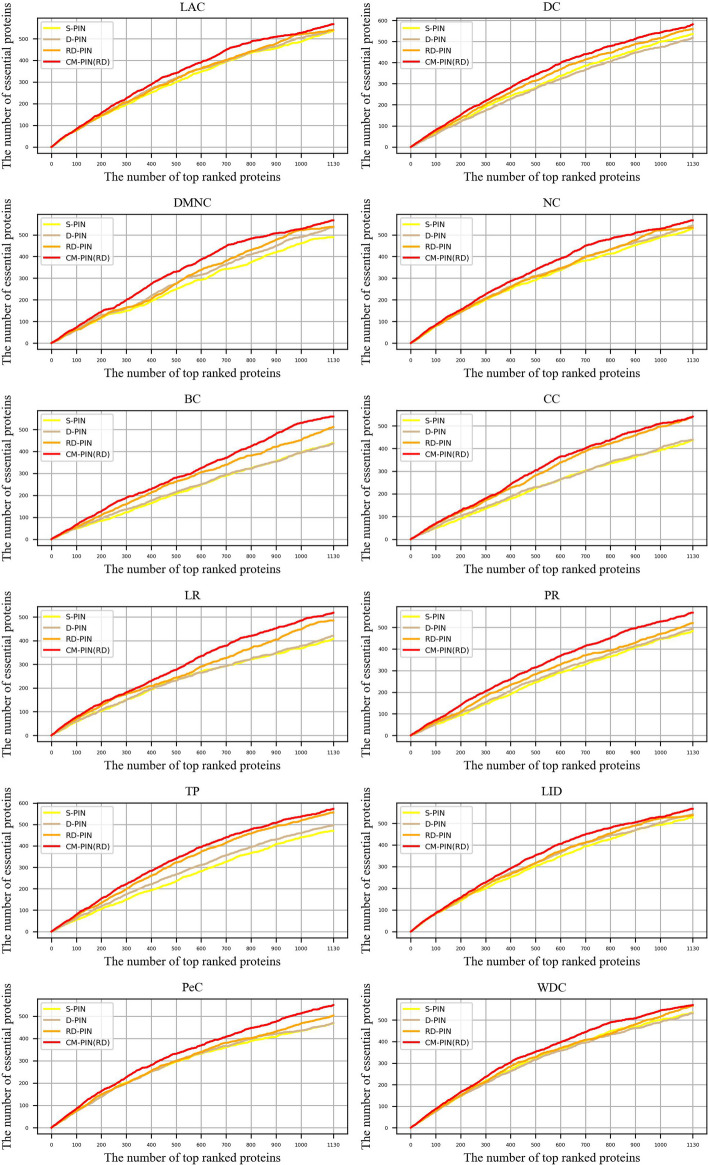
12 node ranking methods are validated by the Jackknife methodology on YDIP dataset

**Fig. 3.**
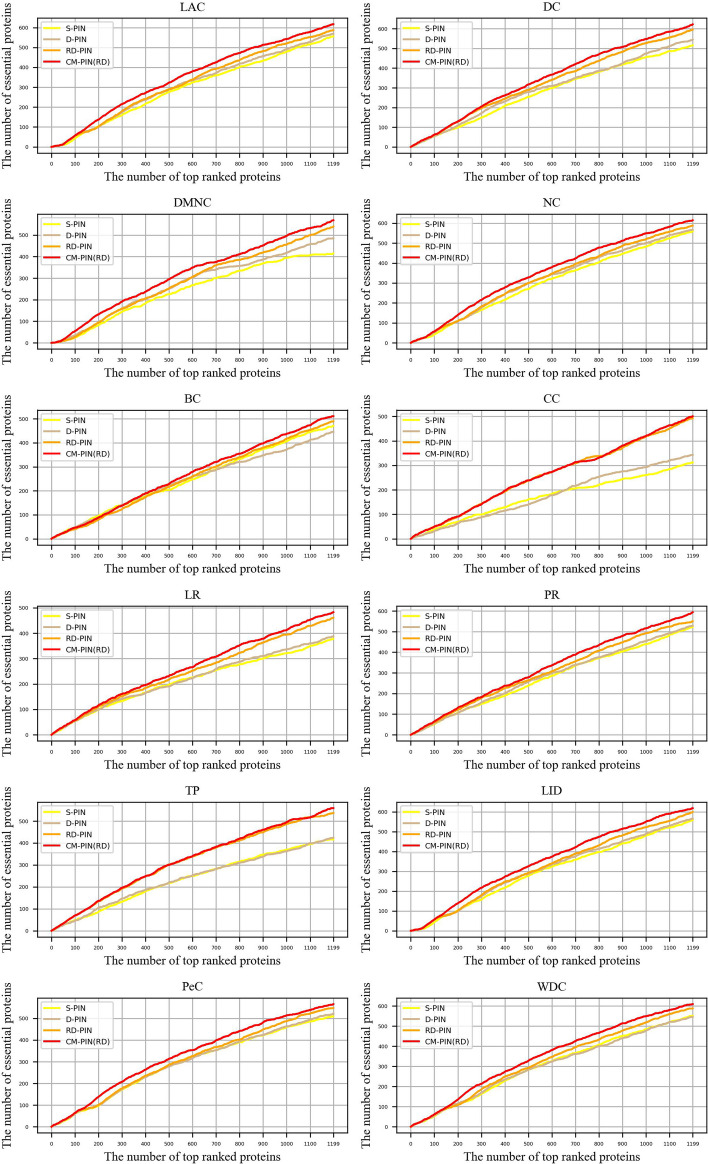
12 node ranking methods are validated by the Jackknife methodology on YBioGRID dataset

In order to evaluate the overall performance of CM-PIN more comprehensively, we used the Jackknifing method [24, 54]. The horizontal axis of the Jackknifing plot indicates the number of proteins that ranked high in the network and the vertical axis represents the number of essential proteins among these top-ranked proteins. Figures 2 and 3 showed the number of essential proteins in the top K highest scoring proteins for each node ranking method in S-PIN, D-PIN, RD-PIN and CM-PIN (the CM-PIN with the best performance of the node ranking method among the three CM-PINs is selected), Among them, K is the number of essential proteins, K = 1130 and K = 1199 on YDIP and YBioGRID respectively. It is obvious that on the CM-PIN, the Jackknifing curves of these methods are all above the other three networks on both two yeast datasets, and the differences are significant, whether it is neighborhood-based, path-based or eigenvector-based centrality methods, even the node sorting methods that integrates multiple biological information. This further demonstrated that the network refinement method in this paper is effective in removing noise and false positives from protein interaction networks and proved that the CM-PIN is a more efficient network.

#### Analysis of precision-recall curves

**Fig. 4:**
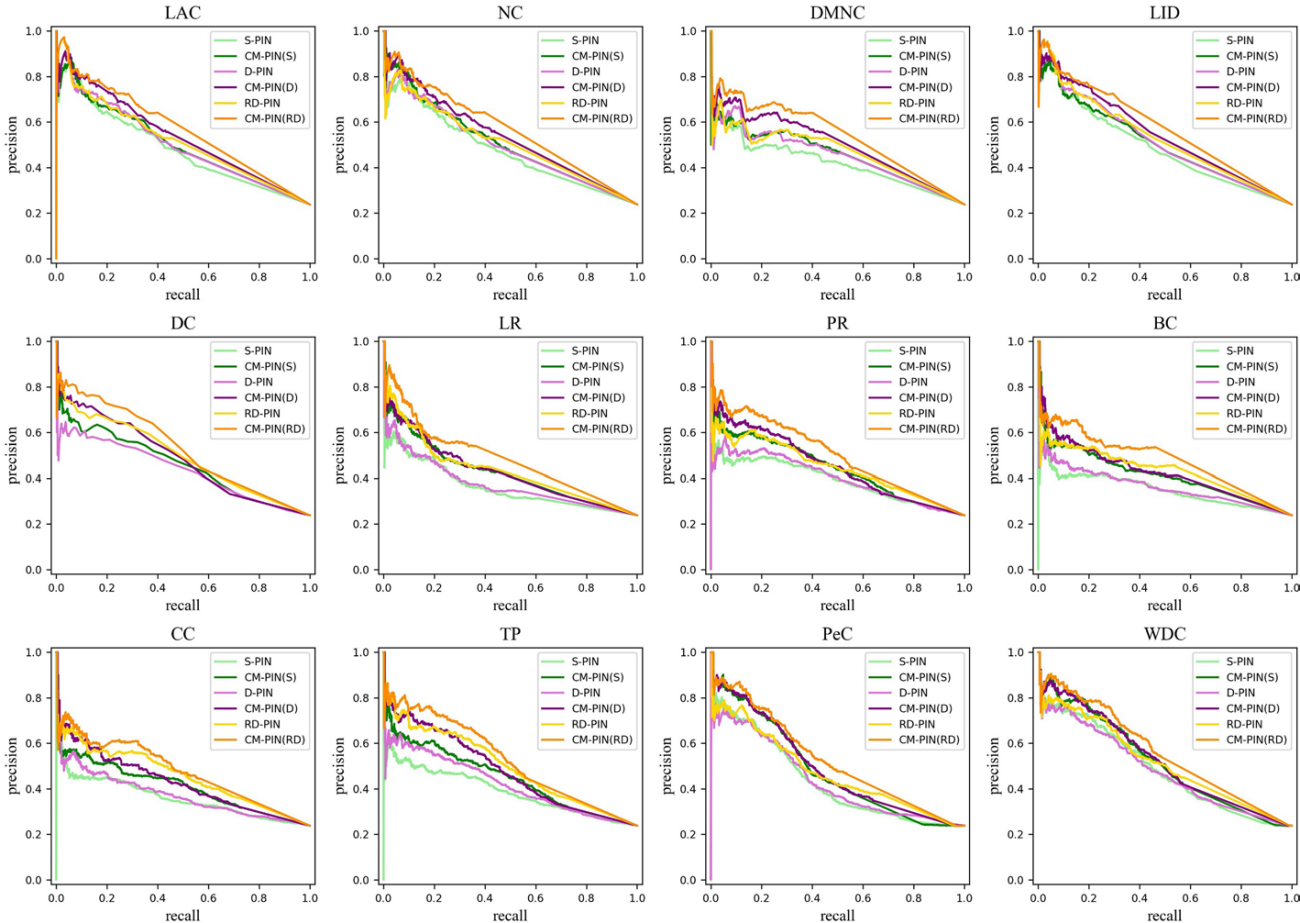
Comparison of precision-recall curves of 12 nodes ranking methods on on YDIP dataset

**Fig. 5:**
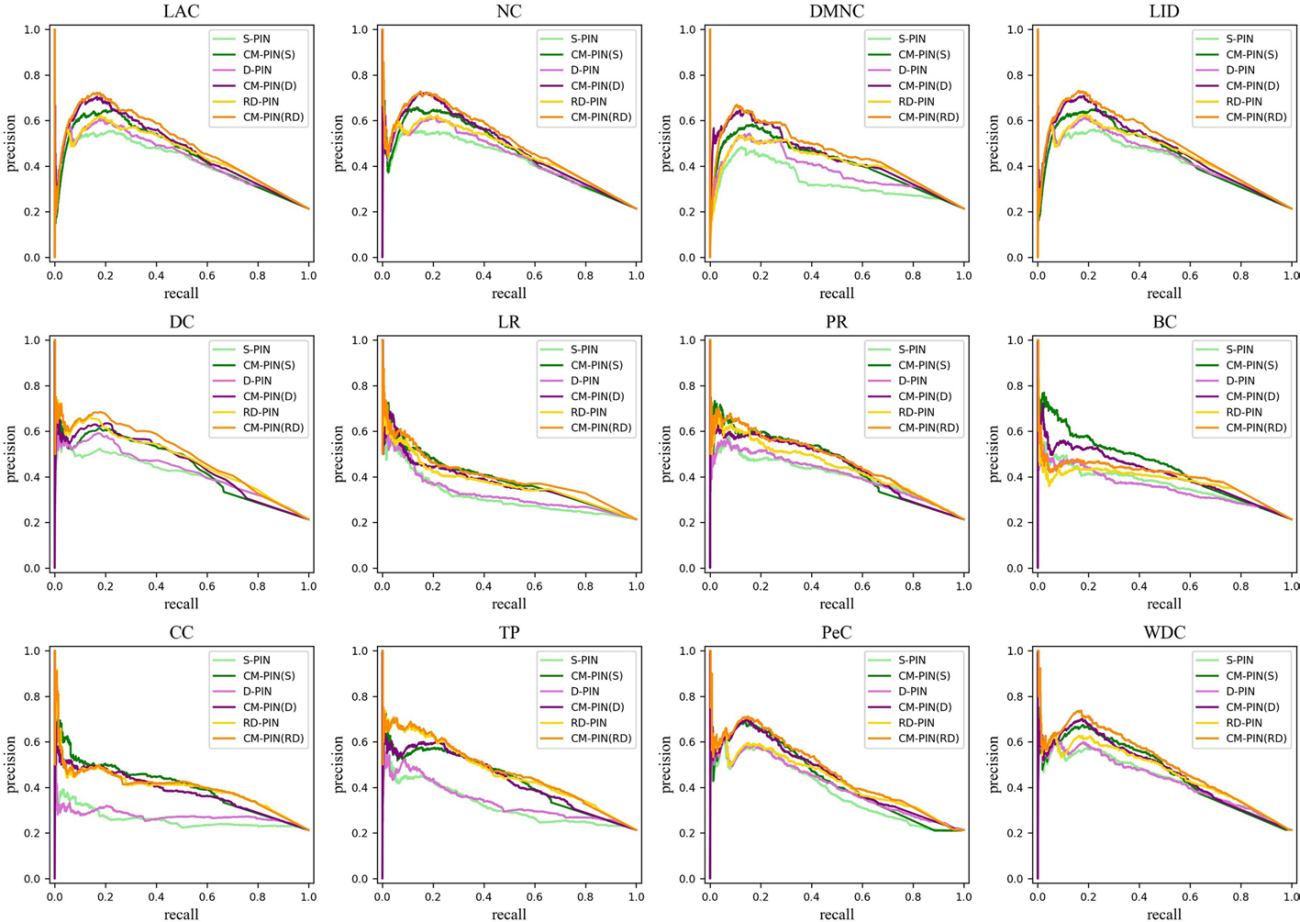
Comparison of precision-recall curves of 12 nodes ranking methods on on YBioGRID dataset

As the identification of essential proteins is a sample imbalance problem, the number of negative class samples (non-essential proteins) is much larger than the number of positive class samples (essential proteins). When it comes to identifying essential proteins, we tend to more concerned with how many positive samples (essential proteins) can be identified [55]. Therefore, to assess the significance of the CM-PIN, we used precision-recall curves to compare the efficiency of essential protein identification of 12 node ranking methods (see Figs. 4 and 5). The vertical axis (precision) of the precision-recall curve reflects the proportion of the true positive examples in the positive examples determined by the classifier, and the horizontal axis (recall) reflects the proportion of the positive examples determined by the classifier in the total positive examples. What’s more, we further calculated the area under the precision-recall curve (PRAUC), as shown in Table 4, and it can be seen that both the precision-recall curves and PRAUC values on the CM-PIN of two yeast datasets were the best. The improvement rate of PRAUC value of 12 node ranking methods on the CM-PIN on YDIP and YBioGRID was: 3.28%-18.29% and 7.18%-54.62% for S-PIN; 5.85%-17.36% and 6.81%-38.55% for D-PIN; 4.61%-15.70% and 0.50%-11.63% for RD-PIN. All of these proved the validity of the CM-PIN again.

**Table 4:** Comparison of PRAUC values of 12 node ranking methods on the S-PIN, D-PIN, RD-PIN and their corresponding CM-PIN on YDIP and YBioGRID datasets

Methods	YDIP			YBioGRID		
	S-PIN/CM-PIN(S)	D-PIN/CM-PIN(D)	RD-PIN/CM-PIN(RD)	S-PIN/CM-PIN(S)	D-PIN/CM-PIN(D)	RD-PIN/CM-PIN(RD)
LAC	0.484/**0.502**	0.503/**0.537**	0.517/**0.567**	0.413/**0.446**	0.431/**0.469**	0.448/**0.489**
DC	0.411/**0.459**	0.435/**0.486**	0.497/**0.530**	0.410/**0.444**	0.426/**0.455**	0.474/**0.494**
DMNC	0.421/**0.454**	0.451/**0.500**	0.465/**0.538**	0.329/**0.413**	0.376/**0.436**	0.404/**0.451**
NC	0.478/**0.506**	0.496/**0.535**	0.506/**0.565**	0.424/**0.459**	0.442/**0.480**	0.461/**0.499**
TP	0.387/**0.448**	0.419/**0.478**	0.497/**0.528**	0.321/**0.429**	0.338/**0.430**	0.463/**0.469**
LID	0.488/**0.504**	0.513/**0.543**	0.528/**0.572**	0.418/**0.448**	0.435/**0.472**	0.451/**0.491**
BC	0.350/**0.414**	0.363/**0.426**	0.433/**0.480**	0.364/**0.442**	0.354/**0.409**	0.379/**0.399**
CC	0.360/**0.411**	0.372/**0.428**	0.456/**0.477**	0.260/**0.402**	0.275/**0.381**	0.398/**0.400**
PR	0.388/**0.443**	0.400/**0.453**	0.445/**0.501**	0.404/**0.452**	0.408/**0.445**	0.442/**0.468**
LR	0.362/**0.423**	0.373/**0.426**	0.433/**0.483**	0.312/**0.389**	0.327/**0.376**	0.374/**0.401**
PeC	0.431/**0.480**	0.435/**0.491**	0.470/**0.526**	0.384/**0.429**	0.406/**0.443**	0.430/**0.466**
WDC	0.486/**0.518**	0.483/**0.526**	0.516/**0.561**	0.430/**0.462**	0.432/**0.466**	0.465/**0.501**

#### Validated by accuracy

To further evaluate the overall performance of CM-PIN and the accuracy of essential protein identification, we used the following seven evaluation metrics: sensitivity (SN), specificity (SP), positive predictive value (PPV), negative predictive value (NPV), F-measure (FM), Matthews correlation coefficient (MCC) and accuracy (ACC). Among them, the calculation formulas of sensitivity and recall are consistent, the calculation formulas of positive predictive value and precision are also consistent. The top K proteins after the descending order of importance scores of proteins were assumed to be essential proteins (K = 1130 and K = 1199 are the number of essential proteins for the YDIP and YBioGRID), and the calculation formulas are as follows,

8 $$SN = \frac{TP}{{TP + FN}}$$

9 $$SP = \frac{TN}{{FP + TN}}$$

10 $$PPV = \frac{TP}{{TP + FP}}$$

11 $$NPV = \frac{TN}{{TN + FN}}$$

12 $$FM = \frac{2 \times SN \times PPV}{{SN + PPV}}$$

13 $$MCC = \frac{TP \times TN - FP \times FN}{{\sqrt {(TP + FP)(TP + FN)(TN + FP)(TN + FN)} }}$$

14 $$ACC = \frac{TP + TN}{{TP + TN + FP + FN}}$$

where *TP* is the correctly predicted essential protein, *FP* stands for the incorrectly predicted essential protein, *TN* refers to the correctly predicted non-essential protein, and *FN* represents the incorrectly predicted non-essential protein.

Tables 5 and 6 showed the comparison results of the 12 node ranking methods on the seven indicators of S-PIN, D-PIN, RD-PIN and CM-PIN (RD). It can be seen that the seven evaluation indicators of the 12 node ranking methods on the CM-PIN on two yeast datasets are both better than the other three networks, which indicates that the method of refining networks by modules in this paper is feasible and can effectively improve the identification accuracy of essential proteins.

**Table 5:** Comparison of seven evaluation indices for 12 node ranking methods on YDIP datasets

Methods	PIN	SN	SP	PPV	NPV	FM	MCC	ACC
LAC	S-PIN	0.474	0.836	0.474	0.836	0.474	0.309	0.749
	D-PIN	0.478	0.837	0.478	0.837	0.478	0.315	0.751
	RD-PIN	0.478	0.837	0.478	0.837	0.478	0.315	0.751
	**CM-PIN(RD)**	**0.502**	**0.844**	**0.502**	**0.844**	**0.502**	**0.346**	**0.763**
DC	S-PIN	0.442	0.826	0.442	0.826	0.442	0.267	0.734
	D-PIN	0.458	0.831	0.458	0.831	0.458	0.289	0.742
	RD-PIN	0.495	0.842	0.495	0.842	0.495	0.337	0.759
	**CM-PIN(RD)**	**0.515**	**0.849**	**0.515**	**0.849**	**0.515**	**0.364**	**0.769**
DMNC	S-PIN	0.433	0.823	0.433	0.823	0.433	0.256	0.730
	D-PIN	0.474	0.836	0.474	0.836	0.474	0.309	0.749
	RD-PIN	0.476	0.836	0.476	0.836	0.476	0.312	0.751
	**CM-PIN(RD)**	**0.502**	**0.844**	**0.502**	**0.844**	**0.502**	**0.346**	**0.763**
NC	S-PIN	0.468	0.834	0.468	0.834	0.468	0.302	0.747
	D-PIN	0.481	0.838	0.481	0.838	0.481	0.318	0.753
	RD-PIN	0.472	0.835	0.472	0.835	0.472	0.307	0.748
	**CM-PIN(RD)**	**0.502**	**0.844**	**0.502**	**0.844**	**0.502**	**0.346**	**0.763**
TP	S-PIN	0.416	0.818	0.416	0.818	0.416	0.233	0.722
	D-PIN	0.439	0.825	0.439	0.825	0.439	0.264	0.733
	RD-PIN	0.492	0.841	0.492	0.841	0.492	0.333	0.758
	**CM-PIN(RD)**	**0.507**	**0.846**	**0.507**	**0.846**	**0.507**	**0.353**	**0.765**
LID	S-PIN	0.467	0.834	0.467	0.834	0.467	0.301	0.746
	D-PIN	0.478	0.837	0.478	0.837	0.478	0.315	0.751
	RD-PIN	0.476	0.836	0.476	0.836	0.476	0.312	0.751
	**CM-PIN(RD)**	**0.502**	**0.844**	**0.502**	**0.844**	**0.502**	**0.346**	**0.763**
BC	S-PIN	0.389	0.809	0.389	0.809	0.389	0.197	0.709
	D-PIN	0.386	0.808	0.386	0.808	0.386	0.194	0.708
	RD-PIN	0.452	0.829	0.452	0.829	0.452	0.281	0.739
	**CM-PIN(RD)**	**0.495**	**0.842**	**0.495**	**0.842**	**0.495**	**0.337**	**0.759**
CC	S-PIN	0.386	0.808	0.386	0.808	0.386	0.194	0.708
	D-PIN	0.389	0.809	0.389	0.809	0.389	0.197	0.709
	RD-PIN	0.478	0.837	0.478	0.837	0.478	0.315	0.751
	**CM-PIN(RD)**	**0.479**	**0.837**	**0.479**	**0.837**	**0.479**	**0.316**	**0.752**
PR	S-PIN	0.427	0.821	0.427	0.821	0.427	0.249	0.727
	D-PIN	0.436	0.824	0.436	0.824	0.436	0.260	0.732
	RD-PIN	0.460	0.831	0.460	0.831	0.460	0.292	0.743
	**CM-PIN(RD)**	**0.503**	**0.845**	**0.503**	**0.845**	**0.503**	**0.347**	**0.763**
LR	S-PIN	0.361	0.800	0.361	0.800	0.361	0.161	0.696
	D-PIN	0.372	0.804	0.372	0.804	0.372	0.175	0.701
	RD-PIN	0.429	0.822	0.429	0.822	0.429	0.251	0.728
	**CM-PIN(RD)**	**0.458**	**0.831**	**0.458**	**0.831**	**0.458**	**0.289**	**0.742**
PeC	S-PIN	0.413	0.817	0.413	0.817	0.413	0.230	0.721
	D-PIN	0.415	0.817	0.415	0.817	0.415	0.232	0.721
	RD-PIN	0.445	0.827	0.445	0.827	0.445	0.272	0.736
	**CM-PIN(RD)**	**0.487**	**0.840**	**0.487**	**0.840**	**0.487**	**0.326**	**0.756**
WDC	S-PIN	0.473	0.835	0.473	0.835	0.473	0.309	0.749
	D-PIN	0.471	0.835	0.471	0.835	0.471	0.305	0.748
	RD-PIN	0.501	0.844	0.501	0.844	0.501	0.345	0.762
	**CM-PIN(RD)**	**0.504**	**0.845**	**0.504**	**0.845**	**0.504**	**0.350**	**0.764**

**Table 6:** Comparison of seven evaluation indices for 12 node ranking methods on YBIOGRID datasets

Methods	PIN	SN	SP	PPV	NPV	FM	MCC	ACC
LAC	S-PIN	0.464	0.854	0.464	0.854	0.464	0.318	0.771
	D-PIN	0.475	0.857	0.475	0.857	0.475	0.332	0.776
	RD-PIN	0.490	0.862	0.490	0.862	0.490	0.352	0.782
	**CM-PIN(RD)**	**0.515**	**0.868**	**0.515**	**0.868**	**0.515**	**0.383**	**0.793**
DC	S-PIN	0.430	0.845	0.430	0.845	0.430	0.276	0.757
	D-PIN	0.455	0.852	0.455	0.852	0.455	0.307	0.767
	RD-PIN	0.497	0.863	0.497	0.863	0.497	0.361	0.785
	**CM-PIN(RD)**	**0.520**	**0.870**	**0.520**	**0.870**	**0.520**	**0.389**	**0.795**
DMNC	S-PIN	0.346	0.823	0.346	0.823	0.346	0.169	0.721
	D-PIN	0.405	0.839	0.405	0.839	0.405	0.244	0.746
	RD-PIN	0.450	0.851	0.450	0.851	0.450	0.301	0.765
	**CM-PIN(RD)**	**0.475**	**0.857**	**0.475**	**0.857**	**0.475**	**0.332**	**0.776**
NC	S-PIN	0.467	0.855	0.467	0.855	0.467	0.322	0.772
	D-PIN	0.473	0.857	0.473	0.857	0.473	0.330	0.775
	RD-PIN	0.490	0.862	0.490	0.862	0.490	0.352	0.782
	**CM-PIN(RD)**	**0.513**	**0.868**	**0.513**	**0.868**	**0.513**	**0.381**	**0.792**
TP	S-PIN	0.347	0.823	0.347	0.823	0.347	0.170	0.721
	D-PIN	0.354	0.825	0.354	0.825	0.354	0.178	0.724
	RD-PIN	0.448	0.850	0.448	0.850	0.448	0.298	0.764
	**CM-PIN(RD)**	**0.466**	**0.855**	**0.466**	**0.855**	**0.466**	**0.321**	**0.772**
LID	S-PIN	0.466	0.855	0.466	0.855	0.466	0.321	0.772
	D-PIN	0.472	0.857	0.472	0.857	0.472	0.329	0.775
	RD-PIN	0.500	0.864	0.500	0.864	0.500	0.365	0.787
	**CM-PIN(RD)**	**0.516**	**0.869**	**0.516**	**0.869**	**0.516**	**0.385**	**0.793**
BC	S-PIN	0.393	0.835	0.393	0.835	0.393	0.228	0.741
	D-PIN	0.372	0.830	0.372	0.830	0.372	0.202	0.732
	RD-PIN	0.409	0.839	0.409	0.839	0.409	0.248	0.748
	**CM-PIN(RD)**	**0.426**	**0.844**	**0.426**	**0.844**	**0.426**	**0.270**	**0.755**
CC	S-PIN	0.260	0.799	0.260	0.799	0.260	0.059	0.684
	D-PIN	0.286	0.806	0.286	0.806	0.286	0.092	0.695
	RD-PIN	0.410	0.840	0.410	0.840	0.410	0.250	0.748
	**CM-PIN(RD)**	**0.418**	**0.842**	**0.418**	**0.842**	**0.418**	**0.260**	**0.751**
PR	S-PIN	0.435	0.847	0.435	0.847	0.435	0.282	0.759
	D-PIN	0.440	0.848	0.440	0.848	0.440	0.289	0.761
	RD-PIN	0.459	0.853	0.459	0.853	0.459	0.312	0.769
	**CM-PIN(RD)**	**0.495**	**0.863**	**0.495**	**0.863**	**0.495**	**0.358**	**0.785**
LR	S-PIN	0.315	0.814	0.315	0.814	0.315	0.129	0.708
	D-PIN	0.324	0.816	0.324	0.816	0.324	0.140	0.711
	RD-PIN	0.384	0.833	0.384	0.833	0.384	0.217	0.737
	**CM-PIN(RD)**	**0.403**	**0.838**	**0.403**	**0.838**	**0.403**	**0.241**	**0.745**
PeC	S-PIN	0.425	0.844	0.425	0.844	0.425	0.268	0.754
	D-PIN	0.435	0.847	0.435	0.847	0.435	0.282	0.759
	RD-PIN	0.457	0.853	0.457	0.853	0.457	0.310	0.768
	**CM-PIN(RD)**	**0.472**	**0.857**	**0.472**	**0.857**	**0.472**	**0.329**	**0.775**
WDC	S-PIN	0.460	0.853	0.460	0.853	0.460	0.313	0.769
	D-PIN	0.455	0.852	0.455	0.852	0.455	0.308	0.767
	RD-PIN	0.490	0.862	0.490	0.862	0.490	0.352	0.782
	**CM-PIN(RD)**	**0.508**	**0.866**	**0.508**	**0.866**	**0.508**	**0.374**	**0.790**

### Selection and analysis of thresholds

In this section, taking the RD-PIN of the YDIP as an example, first, we described the concrete steps of construction of the CM-PIN on the basis of the RD-PIN and the motivation of using PIN's modular feature refining network. Then, we analyzed how to select the thresholds. Finally, we listed the thresholds used by all the CM-PINs built on the two yeast datasets in this paper.

On YDIP dataset, the optimal partitioning of modules was achieved by the Fast-unfolding algorithm when the modularity *Q* = 0.7408, at which point the RD-PIN was partitioned into 26 modules. We calculated three metrics for each module in RD-PIN: *PC*, *NSL*, and *TF* (as shown in Table 7) by using the biological information of the proteins and the topological information of the modules in the network. We also observed the number and proportion of essential proteins in each module and found that there was variation between modules and that some modules with sparse interactions within modules or with little biologically important information contained few essential proteins, which may be the potential non-critical modules. For example, the *NSL* values of modules 1, 24, and 26 are zero, which means that the proteins in their modules do not appear in the subcellular compartments of the nucleus, and after the thresholds screening, they will likely be defined as non-critical modules. Therefore, in order to get a more effective network, we need to try to identify seemingly more critical modules in the network and remove some of the interactions in modules with less biological and topological information.

**Table 7:** Biological and topological characterization of each module in the RD-PIN on YDIP dataset

Modules	Corr	NSL	TF	Number of proteins/essential proteins
1	−0.0258	0	1.6667	33/3
2	−0.0764	2.1847	0.4775	222/78
3	−0.0075	0.1563	0.875	32/5
4	−0.023	0.925	1.125	40/7
5	0.1688	3.12	2.68	175/128
6	−0.0314	1.9419	0.2674	86/28
7	−0.016	1.4701	0.3846	117/27
8	0.0362	2.2596	0.5096	104/40
9	−0.0684	2.5321	0.9423	156/50
10	−0.0358	0.4701	1.3806	134/33
11	0.1013	0.0467	1.6822	107/42
12	0.0317	2.8403	1.6736	144/76
13	−0.001	2.2963	0.1481	27/9
14	0.0824	2.01	1.33	100/43
15	−0.0314	2.4423	0.8077	52/18
16	0.0074	0.1111	2.5556	9/6
17	−0.0493	2.3765	0.6	85/39
18	0.0612	2.8462	1.2051	78/38
19	0.0609	0.0962	1.1731	52/15
20	−0.0736	2.8298	0.2128	47/14
21	−0.0989	2.0381	0.8095	105/45
22	0.0264	1.9286	1	28/15
23	−0.0208	1.6	0.4	20/1
24	−0.0088	0	1.3929	28/2
25	−0.0543	0.0857	1.8	35/1
26	−0.0459	0	0.8333	6/0

To obtain the variation rule of the effect of thresholds on the selection of critical modules and the performance of the network, according to the data distribution of three metrics in the module, we let *th*_*1*_ ∈ {−0.02, −0.005, 0.015}, *th*_*2*_ ∈ {1.5, 2}, *th*_*3*_ ∈ {0.25, 0.5}, and listed the effect of the networks on the identification accuracy of essential proteins with different values of the thresholds, respectively (as shown in Table 8, the experimental results in the table are the performance of LID in different networks). The experimental results showed that when *th*_*1*_ and *th*_*2*_ were small and *th*_*3*_ was large, more critical modules were selected. At this time, there was still a large amount of noise in the network that had not been eliminated and the improvement in identification accuracy of essential proteins was not significant, for example, when *th*_*1*_ = −0.02, *th*_*2*_ = 1.5 and *th*_*3*_ = 0.5, the identification accuracy of essential proteins at top 600 and PRAUC have improved compared with RD-PIN, but the identification accuracy of essential proteins at top 1130 is not as good as RD-PIN. In contrast, when *th*_*1*_ and *th*_*2*_ were larger, fewer critical modules were selected. At this time, critical parts of the network may have been removed, and the improvement in the network's identification accuracy of essential proteins was not optimal, for example, when *th*_*1*_ = 0.015, *th*_*2*_ = 2 and *th*_*3*_ = 0.5, the identification accuracy of essential proteins at top 1130 of LID in CM-PIN was still inferior to RD-PIN. Among them, the change of *th*_*1*_ and *th*_*2*_ has a greater impact on the selection of modules, because biological information can better assist in identifying essential proteins than the topology information of the network. When *th*_*1*_ = −0.005, *th*_*2*_ = 2 and *th*_*3*_ = 0.25, the optimal CM-PIN on YDIP dataset is obtained.

**Table 8:** The variation of the effect of thresholds on the selection of critical modules and the performance of the network

*th*_*1*_	*th*_*2*_	*th*_*3*_	Number of critical modules	Top 100, 600,1130	ACC	PRAUC
0.015	1.5	0.25	15	84, 398, 563	0.761	0.564
0.015	2	0.25	13	84, 398, 551	0.756	0.569
0.015	1.5	0.5	13	85, 387, 532	0.748	0.555
0.015	2	0.5	15	83, 383, 533	0.748	0.550
−0.005	1.5	0.25	17	85, 396, 567	0.763	0.567
−**0.005**	**2**	**0.25**	**15**	**85, 405, 567**	**0.763**	**0.572**
−0.005	1.5	0.5	15	84, 395, 541	0.752	0.559
−0.005	2	0.5	17	86, 387, 541	0. 752	0.554
−0.02	1.5	0.25	20	84, 384, 556	0.758	0.554
−0.02	2	0.25	18	85, 387, 555	0.758	0.558
−0.02	1.5	0.5	17	85, 382, 531	0.748	0.551
−0.02	2	0.5	19	86, 383, 535	0.749	0.547

Finally, we listed in Table 9 the selection thresholds and module information of CM-PINs constructed in two datasets of yeast in this paper.

**Table 9:** The selection thresholds and module information of CM-PINs constructed in YDIP and YBioGRID datasets

Datasets	PINs	Modularity (*Q*)	Number of modules	Number of critical modules	*th*_*1*_	*th*_*2*_	*th*_*3*_
YDIP	CM-PIN(S)	0.5369	26	12	0	0.96	−0.78
	CM-PIN(D)	0.6335	28	19	0.001	0.96	−0.02
	CM-PIN(RD)	0.7408	26	15	−0.005	2	0.4
YBioGRID	CM-PIN(S)	0.4566	13	6	0	1	−4
	CM-PIN(D)	0.5256	17	12	0	1	−2
	CM-PIN(RD)	0.6532	19	15	−0.015	1	−2

### Analysis of reasons for the improvement of identification accuracy of essential proteins

**Fig. 6:**
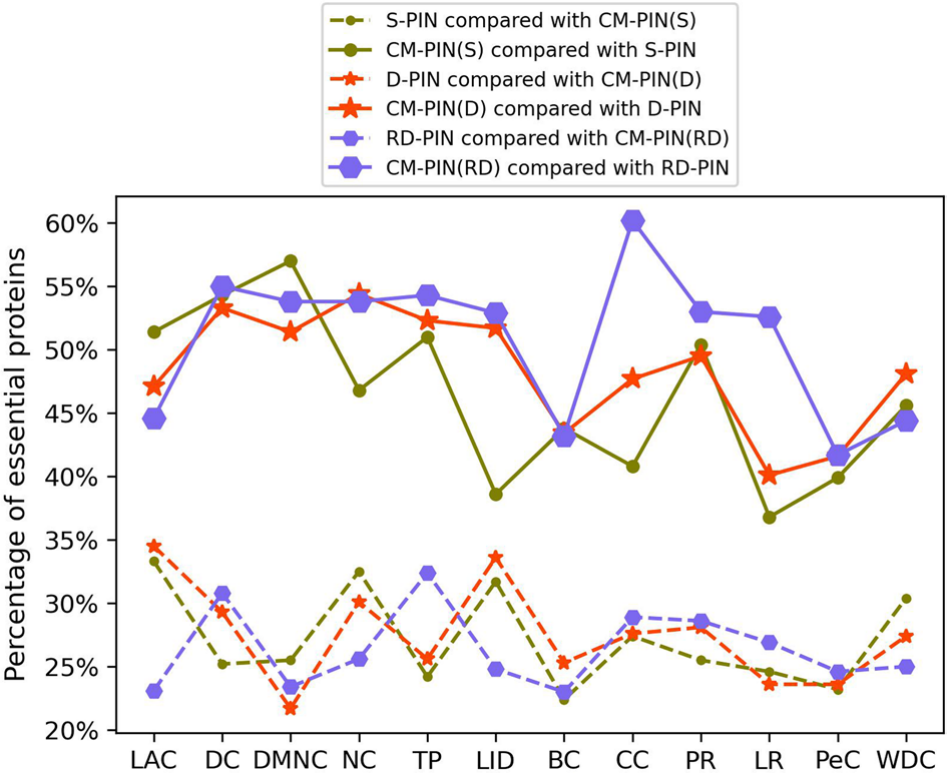
The comparison of the percentage of essential proteins on the CM-PIN with that on the other three networks in different proteins for each node ranking method on YDIP dataset

In order to discuss the reason why the identification accuracy of essential proteins of each node ranking method on the CM-PIN is higher than that on the other three networks (S-PIN, D-PIN, RD-PIN), we also calculated the ratio of essential proteins in different proteins at top 600 of each node ranking method on the CM-PIN and the other three networks, as shown in Fig. 6. It can be seen that on the CM-PIN, each node ranking method can identify some different essential proteins that cannot be identified on the other three networks. Even compared with the best RD-PIN in the three networks, some node ranking methods can identify a large part of different essential proteins at top 600 on the CM-PIN, such as CC, which can identify 31.3% of the different essential proteins on the CM-PIN that cannot be identified on the RD-PIN. Therefore, the essential protein identification accuracy on the CM-PIN is optimal for each node ranking method.

### Validated on Human sapiens

In order to further verify whether the network refinement method proposed in this paper can play its advantages in other species, we obtained their corresponding CM-PINs from S-PIN, D-PIN and RD-PIN in the Human sapiens dataset (Table 10 listed the module information and threshold selection of CM-PINs obtained in each network), and compared the performance of 12 node ranking methods on these networks (see Table 11). It can be seen that the performances of the 12 node ranking methods are almost optimal on the CM-PIN. The performance of the node sorting method on the twice-refined PIN (RD-PIN) is inferior to that on the once-refined PIN (D-PIN) due to fewer raw interactions in the HDIP dataset. That is why the individual indexes of the WDC method on the CM-PIN (refined on the RD-PIN) are inferior to that of the RD-PIN. Compared with S-PIN, D-PIN and RD-PIN, the CM-PINs can improve the PRAUC values of 12 node ranking methods to 14.37%-47.57% for S-PIN, 6.41%-24.90% for D-PIN, and 11.23%-28.11% for RD-PIN. Therefore, this proves that the network refinement method in this paper is applicable to multiple species, and can improve the performance of the node ranking method by obtaining more efficient network CM-PIN.

**Table 10:** The selection thresholds and module information of CM-PINs constructed on HDIP dataset

Datasets	PINs	Modularity (*Q*)	Number of modules	Number of critical modules	*th*_*1*_	*th*_*2*_	*th*_*3*_
HDIP	CM-PIN(S)	0.8069	43	15	0.02	0.4	0
	CM-PIN(D)	0.8095	37	23	0	0.52	0
	CM-PIN(RD)	0.8273	36	21	0.03	0.7	0

**Table 11:** Comparison of various evaluation indicators of 12 node ranking methods on the S-PIN, D-PIN, RD-PIN and the CM-PIN on HDIP dataset (top 100/top 600/MCC/FM/ACC/PRAUC)

Methods	S-PIN	CM-PIN(S)	D-PIN	CM-PIN(D)	RD-PIN	CM-PIN(RD)
LAC	46/204/ 0.189/0.317 0.785/0.296	**59/226/** **0.229/0.350** **0.795/0.357**	44/210/ 0.211/0.335 0.791/0.305	**51/221/** **0.224/0.346** **0.794/0.327**	43/185/ 0.144/0.278 0.773/0.282	**50/204/** **0.199/0.325** **0.788/0.317**
DC	32/158/ 0.122/0.260 0.767/0.236	**48/227/** **0.245/0.364** **0.800/0.311**	33/181/ 0.165/0.296 0.779/0.276	**48/223/** **0.237/0.357** **0.798/0.315**	37/177/ 0.148/0.282 0.774/0.238	**44/199/** **0.202/0.328** **0.789/0.285**
DMNC	43/178/ 0.153/0.287 0.776/0.281	**53/226/** **0.229/0.350** **0.795/0.353**	45/202/ 0.204/0.329 0.789/0.298	**51/224/** **0.247/0.365** **0.800/0.326**	39/186/ 0.148/0.282 0.774/0.282	**48/203/** **0.201/0.326** **0.788/0.316**
NC	47/198/ 0.175/0.304 0.781/0.291	**55/224/** **0.229/0.350** **0.795/0.352**	45/210/ 0.207/0.332 0.790/0.301	**49/223/** **0.233/0.354** **0.797/0.322**	44/184/ 0.140/0.276 0.772/0.275	**49/203/** **0.201/0.326** **0.788/0.310**
TP	29/145/ 0.101/0.242 0.762/0.224	**50/229/** **0.248/0.366** **0.801/0.309**	35/177/ 0.176/0.306 0.782/0.280	**47/226/** **0.250/0.368** **0.801/0.317**	35/176/ 0.152/0.285 0.775/0.240	**43/199/** **0.204/0.329** **0.789/0.284**
LID	49/204/ 0.188/0.315 0.785/0.300	**60/225/** **0.229/0.350** **0.795/0.360**	52/207/ 0.207/0.332 0.790/0.309	**53/224/** **0.242/0.361** **0.799/0.329**	47/181/ 0.134/0.270 0.770/0.285	**48/202/** **0.201/0.326** **0.788/0.317**
BC	23/135/ 0.086/0.230 0.758/0.205	**42/213/** **0.206/0.331** **0.789/0.292**	32/176/ 0.152/0.285 0.775/0.250	**47/208/** **0.224/0.346** **0.794/0.297**	32/153/ 0.109/0.249 0.764/0.222	**36/193/** **0.201/0.326** **0.788/0.272**
CC	19/116/ 0.049/0.198 0.748/0.185	**42/197/** **0.188/0.351** **0.785/0.273**	31/157/ 0.126/0.263 0.768/0.259	**42/212/** **0.211/0.335** **0.791/0.305**	26/134/ 0.099/0.241 0.761/0.219	**29/201/** **0.204/0.329** **0.789/0.263**
PR	26/145/ 0.093/0.236 0.759/0.205	**42/227/** **0.233/0.354** **0.797/0.298**	30/170/ 0.127/0.265 0.769/0.246	**37/210/** **0.229/0.350** **0.795/0.304**	25/148/ 0.072/0.218 0.754/0.217	**41/201/** **0.201/0.326** **0.788/0.278**
LR	23/130/ 0.062/0.200 0.751/0.192	**38/193/** **0.186/0.314** **0.784/0.277**	32/173/ 0.148/0.282 0.774/0.241	**37/212/** **0.214/0.338** **0.792/0.301**	29/161/ 0.148/0.282 0.768/0.223	**37/204/** **0.197/0.324** **0.787/0.279**
PeC	50/231/ 0.230/0.351 0.783/0.327	**56/257/** **0.289/0.401** **0.811/0.374**	50/231/ 0.233/0.354 0.797/0.327	**54/236/** **0.266/0.382** **0.805/0.354**	45/186/ 0.173/0.303 0.781/0.273	**57/206/** **0.181/0.310** **0.783/0.311**
WDC	46/231/ 0.235/0.355 0.797/0.308	**58/245/** **0.238/0.358** **0.798/0.362**	44/230/ 0.227/0.349 0.795/0.312	**55/233/** **0.242/0.361** **0.799/0.332**	44/**190**/ **0.163**/**0.295** **0.778**/0.267	**51**/180/ 0.139/0.274 0.772/**0.300**

## Conclusions and perspectives

In this paper, we proposed a protein interaction network refinement method based on modular discovery and biological information. Firstly, we extract the maximum connected subgraph of a given PIN and use a module discovery algorithm Fast-unfolding to divide it into different modules. Secondly, we select critical modules by using protein orthologous information, subcellular localization information, and its topological information in the PIN. Thirdly, we construct a more refined network (CM-PIN) according to the identified critical modules.

In order to verify the effectiveness of this method, we constructed CM-PINs based on three networks (S-PIN, D-PIN and RD-PIN) of two species (Saccharomyces cerevisiae and Human sapiens) and compared the performances of 12 node ranking methods (LAC, DC, DMNC, NC, TP, LID, CC, BC, PR, LR, PeC, WDC) on the CM-PIN with those on the three networks. In terms of the identification number of essential proteins at top 100- 600, Jackknifing method, the area under the precision-recall curves (PRAUC), sensitivity (SN), specificity (SP), positive predictive value (PPV), negative predictive value (NPV), F-measure (FM), Matthews correlation coefficient (MCC) and accuracy (ACC), the identification performances of node ranking methods on the CM-PIN are better than that of the S-PIN, D-PIN and RD-PIN. Among them, on the three datasets of Saccharomyces cerevisiae (YDIP and YBioGRID) and Human sapiens (HDIP), compared with the existing three networks, the highest improvement rate of PRAUC value of each node ranking method on the CM-PIN was 18.29%, 54.62%, 47.57% for S-PIN; 17.36%, 38.55%, 24.90% for D-PIN; and 15.70%, 11.63%, 28.11% for RD-PIN. The results demonstrated that the CM-PIN could effectively filter out false positives and false negatives and thus is a higher-quality network.

In future work, we will consider further contributing to the identification of essential proteins, the revelation of disease mechanisms and the design of targeted drug from the following three perspectives. Firstly, from the perspective of network refinement, the modular characteristics of the network can be combined with other factors to construct a more efficient network. For example, other biological information of proteins can be used to further refine some unreliable interactions within critical modules, such as structure information or annotation information of proteins. Secondly, from the perspective of module discovery, different module discovery algorithms can attempt to obtain more accurate division results in protein–protein interaction networks, such as clustering algorithms based on biological sequences [56] and attribute graphs [57]. Thirdly, the modules discovered or the critical modules detected from the protein–protein interaction network can also be used as features to assist some other biological issues. For example, the classification task of Golgi protein [58], the classification task of microorganisms’ function proteins [59], design of protein acetylation sites [60], etc.

## Data Availability

The Datasets used in this study, including PINs, gene expression profiles, subcellular localization information, orthologous information, and standard essential proteins, are from the public databases (DIP: http://dip.doe-mbi.ucla.edu; BioGRID: https://thebiogrid.org). The source code for the CM-PIN method and all datasets used in this paper have been uploaded to: https://github.com/paopaopig/The-construction-of-the-CM-PIN..git

## Abbreviations

PIN: Protein–protein interaction network
S-PIN: A network constructed from raw protein–protein interaction dataset
D-PIN: A network refined by S-PIN and gene expression profiles
RD-PIN: A network refined by D-PIN and subcellular localization information
CM-PIN: A refined network based on module discovery and biological information

## References

[1] Winzeler EA, Shoemaker DD, Astromoff A (1999). Functional characterization of the *S. cerevisiae* genome by gene deletion and parallel analysis. Science.

[2] Cullen LM, Arndt GM (2005). Genome-wide screening for gene function using RNAi in mammalian cells. Immunol Cell Biol.

[3] Giaever G, Chu AM, Ni L (2002). Functional profiling of the saccharomyces cerevisiae genome. Nature.

[4] Roemer T, Jiang B (2010). Large-scale essential gene identification in Candida albicans and applications to antifungal drug discovery. Mol Microbiol.

[5] Li X, Li W, Zeng M (2020). Network-based methods for predicting essential genes or proteins: a survey. Brief Bioinform.

[6] Jeong HM, Mason SP, Barabasi AL (2001). Lethality and centrality in protein networks. Nature.

[7] Li M, Wang J, Chen X (2011). A local average connectivity-based method for identifying essential proteins from the network level. Comput Biol Chem.

[8] Wang J, Li M, Wang H (2012). Identification of essential proteins based on edge clustering coefficient. IEEE/ACM Trans Comput Biol Bioinf.

[9] Lin C Y, Chin C H, Wu H H, et al. Hubba: hub objects analyzer—a framework of interactome hubs identification for network biology. Nucleic acids research, 2008, 36(suppl_2): W438–43. 10.1093/nar/gkn257 PMC2447731 18503085

[10] Li M, Lu Y, Wang J, Wu FX, Pan Y. A topology potential-based method for identifying essential proteins from PPI networks. IEEE/ACM Trans Comput Biol Bioinform. 2015 Mar-Apr;12(2):372–83. 10.1109/TCBB.2014.2361350 26357224

[11] Qi Y, Luo J (2015). Prediction of essential proteins based on local interaction density. IEEE/ACM Trans Comput Biol Bioinf.

[12] Wuchty S, Stadler PF (2003). Centers of complex networks. J Theor Biol.

[13] Joy MP, Brock A, Ingber DE (2005). High-betweenness proteins in the yeast protein interaction network. J Biomed Biotechnol.

[14] Brin S, Page L (1998). The anatomy of a large-scale hypertextual Web search engine. Comput. Netw. ISDN Syst..

[15] Lü L, Zhang YC, Yeung CH (2011). Leaders in social networks, the delicious case. PLoS ONE.

[16] Li M, Zhang H, Wang J (2012). A new essential protein discovery method based on the integration of protein–protein interaction and gene expression data. BMC Syst Biol.

[17] Tang X, Wang J, Zhong J (2013). Predicting essential proteins based on weighted degree centrality. IEEE/ACM Trans Comput Biol Bioinf.

[18] Qin C, Sun Y, Dong Y (2016). A new method for identifying essential proteins based on network topology properties and protein complexes. PLoS ONE.

[19] Li M, Lu Y, Niu Z, Wu F (2017). United complex centrality for identification of essential proteins from PPI networks. IEEE/ACM Trans Comput Biol Bioinf.

[20] Lei X, Yang X (2018). A new method for predicting essential proteins based on participation degree in protein complex and subgraph density. PLoS ONE.

[21] Zhong J, Tang C, Peng W (2021). A novel essential protein identification method based on PPI networks and gene expression data. BMC Bioinform.

[22] Von Mering C, Krause R, Snel B (2002). Comparative assessment of large-scale data sets of protein–protein interactions. Nature.

[23] Xiao Q, Wang J, Peng X (2015). Identifying essential proteins from active PPI networks constructed with dynamic gene expression. BMC Genomics BioMed Central.

[24] Li M, Ni P, Chen X (2017). Construction of refined protein interaction network for predicting essential proteins. IEEE/ACM Trans Comput Biol Bioinf.

[25] Meng X, Li W, Peng X (2021). Protein interaction networks: centrality, modularity, dynamics, and applications. Front Comp Sci.

[26] Mitra K, Carvunis AR, Ramesh SK (2013). Integrative approaches for finding modular structure in biological networks. Nat Rev Genet.

[27] Hart GT, Lee I, Marcotte EM (2007). A high-accuracy consensus map of yeast protein complexes reveals modular nature of gene essentiality. BMC Bioinform.

[28] Zotenko E, Mestre J, O'Leary DP (2008). Why do hubs in the yeast protein interaction network tend to be essential: reexamining the connection between the network topology and essentiality. PLoS Comput Biol.

[29] Newman MEJ, Girvan M (2004). Finding and evaluating community structure in networks. Phys Rev E.

[30] Blondel VD, Guillaume JL, Lambiotte R (2008). Fast unfolding of communities in large networks. J Stat Mech: Theory Exp.

[31] Lancichinetti A, Fortunato S (2009). Community detection algorithms: a comparative analysis. Phys Rev E.

[32] Palla G, Derényi I, Farkas I (2005). Uncovering the overlapping community structure of complex networks in nature and society. Nature.

[33] Li M, Meng X, Zheng R (2017). Identification of protein complexes by using a spatial and temporal active protein interaction network. IEEE/ACM Trans Comput Biol Bioinf.

[34] Hu L, Pan X, Tang Z (2021). A fast fuzzy clustering algorithm for complex networks via a generalized momentum method. IEEE Trans Fuzzy Syst.

[35] Hu L, Yang Y, Tang Z, et al. FCAN-MOPSO: an improved fuzzy-based graph clustering algorithm for complex networks with multi-objective particle swarm optimization. IEEE Trans Fuzzy Syst. 2023.

[36] Yang Y, Su X, Zhao B, et al. Fuzzy-based deep attributed graph clustering. IEEE Trans. Fuzzy Syst. 2023.

[37] Zhang Z, Ruan J, Gao J (2019). Predicting essential proteins from protein-protein interactions using order statistics. J Theor Biol.

[38] Wang H, Pan L, Sun J (2022). Centrality combination method based on feature selection for protein interaction networks. IEEE Access.

[39] Li B, Pan L, Sun J (2022). A node ranking method based on multiple layers for dynamic protein interaction networks. IEEE Access.

[40] Barabasi AL, Oltvai ZN (2004). Network biology: understanding the cell's functional organization. Nat Rev Genet.

[41] Nacher JC, Hayashida M, Akutsu T (2009). Emergence of scale-free distribution in protein–protein interaction networks based on random selection of interacting domain pairs. Biosystems.

[42] Zhao B, Wang J, Li X (2016). Essential protein discovery based on a combination of modularity and conservatism. Methods.

[43] Salwinski L, Miller CS, Smith AJ (2004). The database of interacting Proteins: 2004 update. Nucleic Acids Res.

[44] Stark C, Breitkreutz B J, Chatr-Aryamontri A, et al. The BioGRID interaction database: 2011 update. Nucleic Acids Res. 2010; 39(suppl_1): D698–D704. 10.1093/nar/gkq1116 PMC3013707 21071413

[45] Schapke J, Tavares A, Recamonde-Mendoza M (2021). Epgat: gene essentiality prediction with graph attention networks. IEEE/ACM Trans Comput Biol Bioinf.

[46] Zhang R, Lin Y. DEG 5.0, a database of essential genes in both prokaryotes and eukaryotes. Nucleic Acids Res. 2009, 37(suppl_1): D455–D458. 10.1093/nar/gkn858 PMC2686491 18974178

[47] Mewes HW, Frishman D, Mayer K F X, et al. MIPS: analysis and annotation of proteins from whole genomes in 2005. Nucleic acids Res. 2006;34(suppl_1): D169–172. 10.1093/nar/gkj148 PMC1347510 16381839

[48] Chen W H, Lu G, Chen X, et al. OGEE v2: an update of the online gene essentiality database with special focus on differentially essential genes in human cancer cell lines. Nucleic Acids Res. 2016: gkw1013. 10.1093/nar/gkw1013 PMC5210522 27799467

[49] Tu BP, Andrzej K, Maga R (2005). Logic of the yeast metabolic cycle: temporal compartmentalization of cellular processes. Science.

[50] Aran D, Camarda R, Odegaard J (2017). Comprehensive analysis of normal adjacent to tumor transcriptomes. Nat Commun.

[51] Binder J X, Pletscher-Frankild S, Tsafou K, et al. COMPARTMENTS: unification and visualization of protein subcellular localization evidence. Database, 2014. 10.1093/database/bau012 PMC3935310 24573882

[52] Östlund G, Schmitt T, Forslund K, et al. InParanoid 7: new algorithms and tools for eukaryotic orthology analysis. Nucleic Acids Res. 2010;38(suppl_1): D196–D203. 10.1093/nar/gkp931 PMC2808972 19892828

[53] Sonnhammer ELL, Östlund G (2015). InParanoid 8: orthology analysis between 273 proteomes, mostly eukaryotic. Nucleic Acids Res.

[54] Holman AG, Davis PJ, Foster JM (2009). Computational prediction of essential genes in an unculturable endosymbiotic bacterium, Wolbachia of Brugia malayi. BMC Microbiol.

[55] Meng X, Li W, Xiang J (2022). Temporal-spatial analysis of the essentiality of hub proteins in protein-protein interaction networks. IEEE Trans Netw Sci Eng.

[56] Li G, Zhao B, Su X, et al. Discovering consensus regions for interpretable identification of rna n6-methyladenosine modification sites via graph contrastive clustering. IEEE J Biomed Health Inform. 2024. 10.1109/JBHI.2024.3357979 38265898

[57] Hu L, Pan X, Yan H (2021). Exploiting higher-order patterns for community detection in attributed graphs. Integr Comput-Aided Eng.

[58] Bao W, Gu Y, Chen B (2023). Golgi_DF: golgi proteins classification with deep forest. Front Neurosci.

[59] Bao W, Liu Y, Chen B (2024). Oral_voting_transfer: classification of oral microorganisms’ function proteins with voting transfer model. Front Microbiol.

[60] Bao W, Yang B (2024). Protein acetylation sites with complex-valued polynomial model. Front Comput Sci.

